# The feasibility of negative pressure wound therapy versus standard dressings in paediatric hand and foot burns protocol: a pilot, single-centre, randomised control trial

**DOI:** 10.1186/s40814-023-01308-z

**Published:** 2023-05-26

**Authors:** Emma Lumsden, Roy Kimble, Catherine McMillan, Kristen Storey, Robert S. Ware, Bronwyn Griffin

**Affiliations:** 1 Present Address: Faculty of Health, Griffith University, Gold Coast Campus, Parklands Dr, Southport, QLD 4222 Australia; 2 Queensland Children’s Hospital, Stanley St., South Brisbane, QLD 4101 Australia; 3 Centre for Children’s Health Research, 62 Graham Street, South Brisbane, QLD 4101 Australia; 4 Present Address: Menzies Health Institute Queensland, Griffith University, Gold Coast Campus, Parklands Dr, Southport, QLD 4222 Australia

**Keywords:** Hand, Foot, Negative pressure wound therapy, Feasibility and acceptability, Burn, Paediatric

## Abstract

**Introduction:**

The goal of paediatric hand and foot burn management is hypertrophic scar and/or contracture prevention. The risk of scar formation may be minimised by integrating negative pressure wound therapy (NPWT) as an acute care adjunct as it decreases the time to re-epithelialisation. NPWT has known associated therapeutic burden; however, this burden is hypothesised to be outweighed by an increased likelihood of hypertrophic scar prevention. This study will assess the feasibility, acceptability and safety of NPWT in paediatric hand and foot burns with secondary outcomes of time to re-epithelialisation, pain, itch, cost and scar formation.

**Methods and analysis:**

This is a single-site, pilot randomised control trial. Participants must be aged ≤ 16 years, otherwise well and managed within 24 h of sustaining either a hand or foot burn. Thirty participants will be randomised to either standard care (Mepitel®—a silicone wound interface contact dressing—and ACTICOAT™—a nanocrystalline silver-impregnated dressing) or standard care plus NPWT. Patients will be reviewed until 3 months post-burn wound re-epithelialisation, with measurements taken at dressing changes to assess primary and secondary outcomes. Surveys, randomisation and data storage will be done via online platforms and physical data storage collated at the Centre for Children’s Health Research, Brisbane, Australia. Analysis will be performed using the Stata statistical software.

**Ethics and dissemination:**

Queensland Health and Griffith University Human Research ethics approval including a site-specific assessment was obtained. The findings of this study will be disseminated through clinical meetings, conference presentations and peer reviewed journals.

**Trial registration:**

Registered with the Australian and New Zealand Clinical Trials Registry (ACTRN12622000044729, https://www.anzctr.org.au/Trial/Registration/TrialReview.aspx?id=381890&isReview=true, registered 17/01/2022).

**Supplementary Information:**

The online version contains supplementary material available at 10.1186/s40814-023-01308-z.

## Article summary

Strengths and limitations of this study

- Second prospective trial assessing the use of NPWT in paediatric burns
- First randomised control trial to assess the feasibility, acceptability and safety of NPWT in paediatric hand and foot burns
- First trial to attempt to characterise any barriers of the larger NPWT machines
- Pilot trial and consequently small sample size and low power for clinical outcomes
- Trial is based at a single quaternary centre, the generalisability of findings must therefore be interpreted in this context and with care

## Introduction

Paediatric hand and foot burns are common and can have significant therapeutic burden and morbidity [1, 2]. This is often due to hypertrophic scar and/or contracture formation; it is therefore crucial that acute burn care aims to prevent scar development. This may be achieved by reducing the time to re-epithelialisation by integrating the use of negative pressure wound therapy (NPWT) into the acute burn care model.

Isolated hand burns are the most common paediatric burn pattern, particularly in toddlers [3]. This is due to a combination of slow withdrawal reflexes, a desire to explore and a lack of higher order processing, limiting their ability to comprehend the consequences of their actions [4]. Hands are a therapeutically challenging location. Whilst the palmar hand only accounts for approximately 1% total body surface area [5], it facilitates 20 joints [6] and is functionally and cosmetically significant [7]. Paediatric foot burns, although less common, are analogous both anatomically and therapeutically to hand burns and also have a high prevalence in toddlers, who can be unaware of the dangers of hot surfaces [8].

Most hand and foot burns are superficial partial thickness, managed as outpatients and discharged without scar clinic follow-up [9, 10]. However, there is a subcohort with higher associated therapeutic burden and increased risk of morbidity. This is often due to deep, infected or larger burns—all of which decrease the likelihood of scar prevention. Prolonged times to re-epithelialisation, inpatient admissions, operative management, recurrent clinic presentations, scar clinic referrals and years of follow-up are not uncommon trajectories [11, 12]. This is due to hypertrophic scar with or without contracture formation.

A hypertrophic scar is defined as a raised scar above skin level but remains within the margins of injury [13]. These scars usually develop 2 to 3 months post-injury [13] and can be pigmented, hard and thick [14] with a loss of tissue elasticity secondary to an elastin deficiency [15]. An excess of myofibroblast differentiation can ultimately cause tissue contracture [16]. The greatest risk factor for hypertrophic scar formation is prolonged time to wound re-epithelialisation [17]. Risk factors for prolonged re-epithelialisation times include burns that are deeper, more painful, cover larger total body surface areas (TBSA), become infected and have a delayed time to presentation [18]. Hand and foot burns are more painful than burns of other locations [19]. Thus, a deep hand or foot burn with a prolonged time to re-epithelialisation would be considered high risk for hypertrophic scar formation.

Prevention of these scars is imperative as they not only carry a therapeutic burden, they also have significant and sometimes lifelong morbidity [13]. These scars can be functionally restrictive, cosmetically disfiguring, painful and itchy [20]. Fine motor skills may decrease, restricting recreational and vocational pursuits, and self-esteem may drop [21]. Ultimately, these patients may have a decreased quality of life (QoL) with significant psychosocial concerns [22]. One potential way to prevent hypertrophic scar formation is to decrease time to re-epithelialisation through NPWT.

NPWT is a device that optimises wound healing through various mechanisms including establishing a tissue pressure gradient [23], enhancing the wound bed environment, [24] micro- and macro deformations, encouraging cell mitosis [25] and promoting angiogenesis and lymphangiogenesis [26]. Our study team, led by Frear et al., completed a single-site randomised control trial (RCT) of 114 Australian children aged up to 16 years with small, superficial partial thickness paediatric burns and found that NWPT applied within the first 7 days, compared to standard dressings of Mepitel® and ACTICOAT™, decreased the time to re-epithelialisation by 22% (95% CI 7 to 34%) [27]. Through a sub-analysis, it was also identified that when NPWT was applied within the first 24 h, this group re-epithelialised faster than those who had late application [27]. We believe the benefit of NPWT is in modifying the initial progression of the zone of stasis and thus the benefit of NPWT is within the first 72 h. This is also supported by early porcine work in the 1990s [28]. We have therefore altered the application time from our first trial to application occurring within the first 24 h rather than 7 days and removed at the first dressing change—at least 72 h after application without re-application as opposed to when the burn is completely re-epithelialised. However, this study also found that the use of NPWT on paediatric patients had associated burden including the bulk of the device, mechanical issues and difficulties with school attendance. Furthermore, using NPWT on hand and foot burns was often considered inappropriate due to the perceived difficulty of application [27]. Despite this burden, clinicians believe that the burden of NPWT in paediatric hand and foot burns is outweighed by the advantage of decreasing the likelihood of hypertrophic scar formation. We have designed a pilot study to address the specific concerns from the trial by Frear and colleagues, including that hand and foot burns are not a feasible place to apply NPWT and the extent of the burden of the device.

## Methods/design

### Hypothesis and objectives

It is hypothesised that NPWT is feasible, acceptable and safe in paediatric hand and foot burns. Furthermore, it is also hypothesised that by utilising NPWT, burns will have a decreased time to re-epithelialisation and reduced pain, itch, infection and scar formation.

The primary objectives of this pilot study are to determine the feasibility, acceptability and safety of the use of NPWT in paediatric hand and foot burns. The secondary objectives will include investigating time to re-epithelialisation, pain, itch and scar formation. If further research is required, this pilot will help guide the methodology of a larger RCT. Ultimately, this study may inform the integration of NPWT into routine, acute paediatric burns care.

### Trial design

**Fig. 1:**
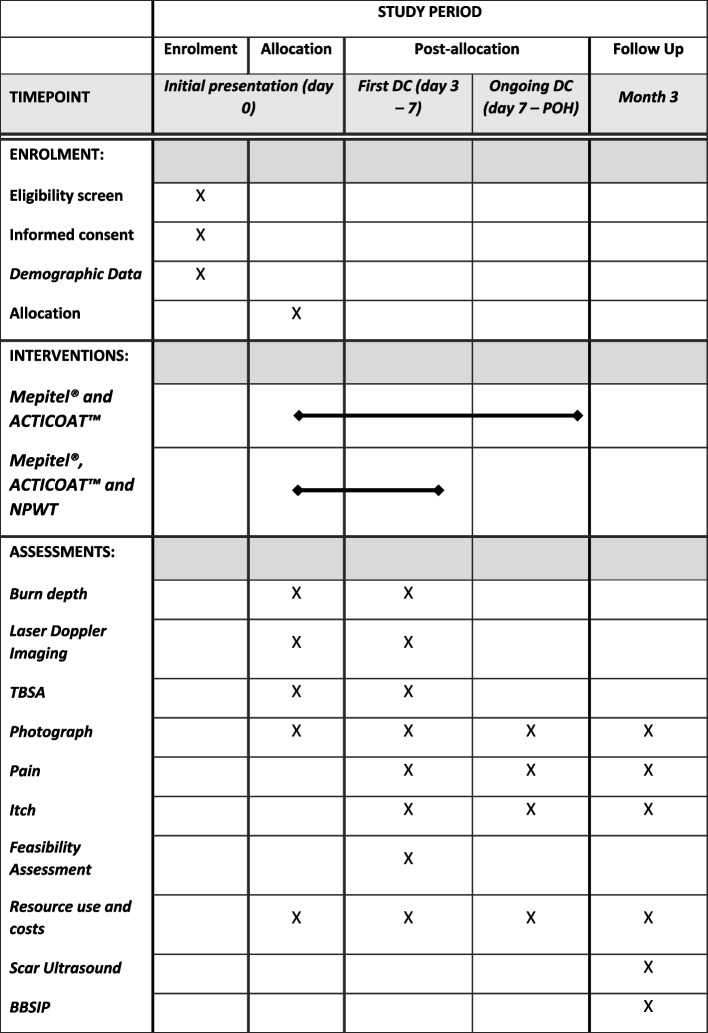
Participant timeline—schedule of enrolment and data collection. DC, dressing change; POH, point of healing; NPWT, negative pressure wound therapy; TBSA, total body surface area; BBSIP, Brisbane Burns Scar Impact Profile

This study is a parallel-group, two-armed, single-centre, pilot, randomised trial contrasting the outcomes of burns patients treated with (1) standard dressings of Mepitel® and ACTICOAT™ (approximately 15 patients, control) against (2) standard dressings plus a NPWT RENASYS TOUCH™ device (approximately 15 patients, intervention). An overview of the study design is summarised in the Standard Protocol Items: Recommendations for Interventional Trials (SPIRIT) figure (Fig. 1) and the SPIRIT checklist (Supplement 1).

### Study setting

This study will be set at the Pegg Leditschke Children’s Burns Centre (PLCBC), Queensland Children’s Hospital (QCH), Brisbane, Australia. QCH is a quaternary specialist paediatric hospital with the burns centre catchment area boundaries including central and southern Queensland and northern New South Wales, an estimated area of 1,053,700 km^2^ with a population of 4.5 million people.

### Eligibility criteria

Participants will include approximately 30 children that are equal to or less than 16 years of age with a burn that is on the hand or foot (may be in the context of a larger burn and the location of the burn on the hand/foot will be recorded), managed within 24 h of sustaining the injury and is either superficial partial thickness and > 0.5% TBSA on one hand or foot or any sized burn that is deep or full thickness. The patient will be excluded if clinician treatment priority contradicts study enrolment, they do not wish to participate, the child is acutely unwell at the time of presentation or consent is unable to be confirmed within 24 h of sustaining the burn injury (Table 1).

**Table 1:** Eligibility criteria

Inclusion criteria	Exclusion criteria
Hand or foot burn, may be in the context of a larger burn	Clinician treatment priority contradicts randomisation
≤ 16 years of age	Do not wish to participate
Managed ≤ 24 h of sustaining a burn injury	Consent unable to be confirmed within 24 h of sustaining burn injury
Superficial partial thickness burn ≥ 0.5% TBSA on one hand or foot	The child is acutely unwell at the time of presentation
Any sized burn that is deep partial or full thickness	TBSA > 10%

### Recruitment/consent

Burns Team and/or Emergency Department (ED) staff will notify researchers of eligible participants presenting to either the ED or the PLCBC. An investigator will provide the patient/family with information (both verbal and written) on the trial, and if the patient/family agrees, they will be asked to sign a consent. Where the participant does not speak English as their first language, an interpreter will be offered. A third party will be present throughout to ensure no coercion and will also sign the consent. If the family does not agree, they will be asked to consent to their baseline demographic data being included. When consenting, it is ensured that the participants/families know they have the autonomy to withdraw from the study at any stage.

Throughout the participants’ treatment, researchers will be independent of any clinical decision making processes regarding the participants. Treating clinicians will decide where the debridement occurs (i.e. theatre, ED, PLCBC) and whether it is appropriate for the patient to be enrolled in the study. Demographic data (i.e. burn details, medical, surgical and social history) will be collected before randomisation into the study.

### Intervention

For all trial patients, appropriate analgesia will be given via either a general anaesthetic or analgesia ± procedural sedation. Next, the burn will be debrided with water and QV Gentle Wash (Ego Pharmaceuticals, Sydney, Australia) and then 10 ml of 5% chlorhexidine gluconate in 500 mL of water. Patients will then have treatment according to the group they were allocated to the following.

#### Control

Participants will receive the standard dressings currently applied at the PLCBC for burns. Mepitel® (Mölnlycke Healthcare, Mikkeli, Finland) and ACTICOAT™ (Smith & Nephew, Hull, UK) will be placed over the burn and secured with Hypafix™ (BSN Medical, Hamburg, Germany). These dressings will by changed every 3 to 7 days (reflective of clinical practice) and continued until the burn is deemed re-epithelialised by the treating clinician. Data will be gathered at each dressing change.

#### Intervention

Participants will include application of the RENASYS TOUCH™ device (Smith & Nephew, Hull, UK) in addition to standard dressings. Kerlix™ AMD Antimicrobial bandage roll (CardinalHealth, Dublin, USA) is first placed as an interface between the standard dressings and NPWT device, then a Suprasorb® CNP EasyDress sleeve (Lohmann & Rauscher, Rengsdorf, Germany) applied over the standard dressing + Kerlix™ and secured with the provided film (± additional semi-permeable transparent film dressing as required to achieve seal). A small hole (< 30 mm) will be cut in the Suprasorb® CNP EasyDress sleeve over the site of the burn and a soft port applied over the hole, ensuring the hole in the EasyDress aligns with the hole of the soft port. The soft port is then attached to a cannister which is attached to the RENASYS TOUCH™ NPWT device. Suction will be applied continuously at either 80 mmHg for children > 1 years old, 60 mmHg for 6 months–1 year or 40 mmHg < 6 months old. If bilateral hands and/or feet are involved, the deeper one will have NPWT applied. If both are assessed to be of equal depth, then NPWT with a y connector will be used. The NPWT will remain on for the first 3–7 days and then removed at the first dressing change; it will not be reapplied after this. Once the NPWT is removed, treatment will continue as per the control group with standard dressings.

Throughout the trial, strategies to improve NPWT adherence will include a backpack to carry the device, dressing packs on discharge that include extra tape to help seal any leaks, education (both written and verbal) on the device and where to call to help trouble shoot issues on discharge.

Criteria for discontinuing or modifying allocated interventions for a given trial participant will include the following: on debridement of the burn, it no longer meets the eligibility criteria (i.e. burn is actually only SPT and is < 0.5% TBSA) or there is an adverse outcome requiring at least admission to the paediatric intensive care unit (PICU). Patients who become septic but are not admitted to PICU or require grafting will remain in the study.

Relevant concomitant care and interventions will include patients in the standard group receiving occupational therapy input including potential splinting from initial presentation. If required, patients in the NPWT group will only commence splinting once the NPWT is removed. The hand will be positioned in a neutral position whilst the NPWT is applied. Kerlix™, an antimicrobial crepe gauze, will aid in maintaining this position and splinting the wound whilst the NPWT is applied.

### Assignment of interventions (randomisation)

Sequence generation will be done via a computer, automated, centralised, randomisation program hosted by the Griffith Randomisation Service. The participants will be in randomised in permutated blocks of sizes two and four so the primary investigator cannot predict the allocation sequence. The participants will be stratified based on whether they are a hand vs foot burn or whether they are being debrided in theatre vs ED. Both recruitment and randomisation will be done by the primary investigator. Once the investigator has consented the patient, they will log onto the service, enter the stratification details and a participant ID and then the group allocation will be generated.

### Blinding

Due to the nature of the study, double blinding will not be possible as both the treating clinicians and the participant will be aware of the treatment modality. Even if NPWT were to be applied to all participants but blinded to whether the intervention has been turned on, it is evident when looking at the dressing what treatment group the participant is in. It will be possible however to blind the clinicians to some of the secondary outcomes. Time to re-epithelialisation will be calculated based on when a blinded panel of clinicians deem photographs of the wound to be 95% re-epithelialised. Similarly, scar thickness will be measured based on three blinded ultrasound photographs. In addition, to minimise bias based on observation of wound re-epithelialisation, any surveys or questions regarding the patient’s progression will be asked of staff/patients before the dressings are removed.

### Ancillary and post-trial care

Standard post-acute care including routine, multi-disciplinary PLCBC scar follow-up will be done.

### Outcomes

#### Pilot trial primary outcomes

Previous acute paediatric burn RCTs have shown that theoretical frameworks for feasibility are often unachievable [27, 29–32]. Given the specificity of the type of burn (significant hand and/or foot burns), it is likely that eligible participants will form a small subcohort. In addition, dropout rates may be higher for the 3-month follow-up especially if there is no scar and the only reason to attend the appointment is for research purposes. Therefore, for this study, a modified feasibility framework has been developed based on the guidelines of Polit and Beck [33] (Table 2). If at the completion of this trial the results are inconclusive or have raised further questions, this study will help guide the feasibility parameters of a larger study.

**Table 2:** Study feasibility parameters

Nil minimum screening rate
Greater than or equal to 80% of eligible participants will agree to enrol
Greater than 80% of participants in the intervention groups will receive their allocated treatment (measured twice weekly whilst participant recruited to study)
Less than 10% of data collection for primary outcomes will be missing
Less than or equal to 40% of participants will be lost to follow-up, withdraw from the study or be deemed ineligible after they have commenced treatment

### Intervention primary outcomes

Questionnaires will be administered to obtain qualitative and quantitative data regarding the feasibility, appropriateness and acceptability of staff, parents and participants using NPWT in acute paediatric hand and foot burns.

#### Staff questionnaire

A mixed method questionnaire incorporating Likert scales and open-ended questions will be developed using Weiner and colleagues’ model for implementation studies [34]. The three criteria addressed are acceptability (is it ethically justifiable to use NPWT in paediatric hand and foot burns), appropriateness (the impact of NPWT on wound re-epithelialisation) and feasibility (is it physically possible to implement NPWT). This questionnaire will be asked at the start and end of the trial to see if the perception of NPWT has changed over the study period. In addition, feasibility of both current standard dressings vs NPWT will be asked in a combined Likert scale and open-ended questionnaire at the point of the first dressing change to assess patient-level data.

#### Participant questionnaire

A mixed method questionnaire incorporating Likert scales and open-ended questions will be administered assessing feasibility at the first dressing change. The questions will address the ease/difficulty and confidence of performing, maintaining and adhering to the assigned dressing. This will allow an analysis of whether there is any perceived difference amongst patients between the dressings.

#### Safety/harm

Safety/harm will be classified based on the number and severity of adverse outcomes. The adverse outcome will be graded based on a modified Clavien-Dindo scale [35]. This is an objective way to quantify the severity of an event from grade one, a deviation from intervention, to grade five, death.

Adverse events will be recorded in an ‘Adverse Outcome Logbook’, stored in a locked cabinet at the Centre for Children’s Health Research. A grade will be given for each day the adverse event occurs. Parents of the participants will be provided with contact details for the burns centre to report any adverse events that may occur during the study. In addition to self-report, parents/participants will be questioned at each clinic appointment through the duration of wound care.

### Secondary outcomes

#### Percentage re-epithelialisation

Clinical photographs will be taken at each dressing change. A panel of experienced burns clinicians will perform a blinded review of the photographs to assess the progress of re-epithelialisation. The burn will be considered healed when 95% re-epithelialised. This method has been used with success in previous studies with primary outcomes of time to re-epithelialisation [27, 36].

#### Pain and distress

Pain and distress will be assessed at home between dressing changes via parents and patients and during dressing changes in clinic via clinicians. Parents and patients will rate their pain based on an age-appropriate scale. Patients aged 3–8 years old will self-report pain intensity using the Faces Pain Scale Revised (FPS-R), a validated scale for paediatric pain assessment. The main benefit is that it demonstrates pain in a gender de-identified face whilst conforming to a linear interval scale that children can relate to [37]. Participants over 8 years old will self-report using the 11-point Pain Numerical Rating Scale (P-NRS) (0 to 10). The P-NRS is validated for children > 8 and is easier to use than the FPS-R as it does not require an additional tool [38]. For children of all ages, parents will report their perceived ranking of their child’s pain on an 11-point P-NRS (0 to 10). There is evidence to suggest good correlation between patient and parent assessment of pain, and it is a useful surrogate when a participant score is not able to be ascertained [39].

Clinicians will assess the child’s pain and distress during dressing changes using the face, legs, activity, cry, consolability (FLACC) scale. This is a validated tool for clinicians to assess pain, with evidence suggesting that with experienced burns nurses (which account for the nursing staff at QCH) the accuracy increases [40]. In addition, any analgesic and/or sedative medications administered to the participant at each dressing change will be recorded. This is given in accordance with the pain ladder, where burns that are likely to be larger will receive stronger medication.

#### Itch

Itch will be assessed by parents, participants and clinicians. Parents will rank the perceived itch for children under 5 years using The Toronto Paediatric Itch Scale—a validated, observation-based scale rating itch behaviours on a scale of 0 (absence of itch) to 4 (severe itch with significant disruption). It helps parents to accurately identify itch based on common paediatric itch patterns rather than the patient having to state they are itchy [41]. Parents of children over five will rate their itch on an 11-point Itch Numeric Rating Scale (I-NRS) (0 to 10). Participants aged 5 to 8 years of age will use the validated Itch Man Scale [42], asking patients to identify which picture on a 5-point scale (0–4) best represents their itch. For children over 8 years, a self-reported 11-point I-NRS (0 to 10) will be used. Clinicians will also be asked to rate the perceived itch on an 11-point I-NRS (0 to 10). The requirement for antipruritic medication will be recorded including the dose and number.

#### Scar/skin assessment (at 3 months)

At 3 months following full re-epithelialisation of the burn injury, a face-to-face follow-up will be completed with all participants to conduct a skin and/or scar review. This time point was picked to assess the development of hypertrophic scar formation, requiring 2–3 months [13]. No surgical interventions for scar management will take place between the time of re-epithelialisation and this appointment. Any garments, splints or silicone-based gels required in the interim will be recorded. An ultrasound scan, using BT12 Venue 40 MSK (GE Healthcare) will be taken to measure the thickness of the scar. This is a validated tool for measuring scar thickness and has been utilised in previous studies [43]. Three central images of the scar will be taken (as opposed to peripherally on the scar border) to use in a blinded analysis by clinicians to confirm scar thickness. The Brisbane Burn Scar Impact Profile (BBSIP) will be used to measure the intensity and frequency of sensations such as pain, tightness and discomfort as well as health-related QoL specific to burn scars. This tool is validated for measuring the health-related QoL in children with burn scars [32]. Where a scar crosses a joint, range of motion will be assessed [19].

#### Cost analysis

A record of the resources and associated cost (costed at market rate) for each participant will be recorded. This will include treatment costs (e.g. the number of dressing changes, type and size of dressings used, scar therapy products) as well as other burn-related resources (and costs) that may be important to a health service deciding which of the interventions to implement in their burn care model. Number of admissions, length of stay, number of operations and clinic appointments will be recorded and costed at hospital projections (i.e. price of hospital bed per night, hourly theatre rate). Labour time (e.g. occupational therapists, physiotherapist, nurses and surgeons) will be quantified for each patient (on the basis of time duration utilised and number of appointments required) and costed at the relevant state award rates for each respective discipline. This data will be recorded with each presentation to hospital.

### Sample size

This is a pilot study. It is primarily designed to determine whether NPWT is a feasible, acceptable and safe intervention in paediatric hand and foot burns given the apprehension of use in this cohort in our previous trial. This study is not powered to achieve statistical significance for secondary outcomes. This pilot study will enrol 30 participants to determine feasibility, safety and acceptability of the proposed intervention. There is minimal relevant literature to base expected outcomes on; sample size has therefore been based off the recommendations of Whitehead et al. [44], Birkett and Day [45] and Browne [46]. Recruiting 30 participants will enable us to estimate the percentage of eligible participants who enrol to within ± 11% (assuming 90% will enrol), the percentage of intervention group participants who receive treatment to within ± 18% (assuming 87% will receive treatment) and the number lost to follow-up, withdrawn or ineligible to within ± 15% (assuming 20% loss), all with alpha = 0.05.

### Data collection methods

Data will be collected using REDCap (Vanderbilt, TN, USA) database developed questionnaires to gather baseline demographic and clinical details (including mechanism, date, time of injury, time to presentation, vaccination status, medical/surgical history, Fitz-Patrick skin type) and assess pain, itch, feasibility, resource use and cost and scar development (Fig. 1). The E-Burn mobile application in combination with photographs will be used to accurately assess TBSA down to 0.1% which is particularly important given most burns will be small TBSAs [47]. E-burn will be used as it is a validated app for TBSA assessment, reducing the risk of human error [48].

Burn depth will be categorized by both laser Doppler imager (LDI) scans and treating consultant review of photographs (taken using a Fujifilm x-T3 camera with a 60-mm lens) at presentation and the first dressing change (between days 3 and 7). The burn will be defined as either ‘superficial partial thickness only’, ‘mixed depth’ or ‘deep dermal partial thickness only’. The photograph review will be blinded.

### Data management/confidentiality

Each participant involved in the study will be de-identified and allocated a unique identifier. Data will be stored in a de-identified manner either online via REDCap and the Griffith Research Space or onsite in a locked cabinet at the Children’s Centre for Health Research.

### Data analysis

Continuous data will be summarised as either mean (standard deviation) or median (interquartile range) depending on distribution, whilst categorical data will be summarised as frequency (percentage). Feasibility outcomes will be presented descriptively as frequency and percentage. Data will be compared between-groups using linear regression (continuous outcomes), logistic regression (binary outcomes) or Poisson regression (count outcomes). In all cases, treatment group will be included as a fixed effect in the analysis model. Stratification variables will be included as covariables when appropriate. The assumptions for each model will be tested, and if model assumptions are not met, comparisons will be made using non-parametric methods. The effect estimates for each model will be presented as mean difference (continuous outcomes), odds ratio (binary outcomes) and incidence rate ratio (count outcomes), although with 95% confidence intervals. The between-group difference between binary outcomes may also be presented as an absolute difference when appropriate. There will be no formal statistical hypothesis testing.

When the outcome variable is measured repeatedly, for example pain, we will use multilevel mixed effects models to account for the likely non-independent of the repeated measures from each participant. In these situations treatment group and time will be included as fixed effects, with a group-by-time interaction.

An intention-to-treat analysis is preferred as it compares all subjects in the groups to which they were originally randomly assigned (despite withdrawal, treatment failure or cross-over). However, we will also conduct per protocol analyses to assess the potential effect of complying to treatment.

### Qualitative data

For feasibility outcomes, researchers will undertake a brief thematic analysis [49] on open-ended questions built into questionnaires, to draw out important common themes relating to the study question, which will not be directly captured through the quantitative measures. We will use specific qualitative data analysis software, NVivo 12, for additional insight and to help identify trends in unstructured data.

### Trial monitoring

The Human Research Ethics Committee will be contacted of any adverse events. In addition, a notification of any adverse outcomes to an external regulatory board comprised of burns clinicians independent of the trial will be completed to ensure nil modifications or trial cessation is required. Regular team meetings will be held where the principal investigators will review study progress, address pertinent issues and identify further actions to take. The principal investigators will ensure, via this regulatory review process, that data is managed appropriately (i.e. stored in a de-identified fashion) and that appropriate steps are taken with regard to data cleansing and dissemination of results.

### Access to data

De-identified data will be available on request after appropriate ethics approval.

### Patient and public involvement

This study was guided by previous patient experience of NPWT, identifying barriers and trying to optimise or better understand these barriers [27]. The patients email address will be collected at recruitment so the results of the study may be disseminated to the participant.

## Discussion

In 2020, our team published a RCT which showed that the use of NPWT in paediatric burns decreased time to burn re-epithelialisation by 22% (95% CI 7–34%) [27]. It is acknowledged however that the establishment of efficacy does not correspond to intervention implementation.

Implementation literature suggests that after the efficacy of an intervention is established, only 50% result in clinical uptake. Of that 50%, the average time to routine care model integration is 17–20 years [50]. Often the problem in uptake is the clinical context and the barriers and challenges of the proposed home environment rather than a lack of evidence to support the intervention [50].

Throughout our centre’s first RCT, we identified barriers to utilising NPWT including the bulk of the device, the ability to mobilise and attend to routine activities of daily care, mechanical issues, the perceived extra work load for clinicians and the paucity of knowledge on how to appropriately apply the intervention [27]. These barriers are instant challenges and, despite the now known efficacy of NPWT, make it less likely that NPWT would be integrated into routine burn care.

This study is therefore primarily a pilot feasibility study on the barriers to NPWT. This will help characterise the barriers in depth with a hope to modify and/or minimise them moving forward. The results of this study will help guide the study design and development of a larger national implementation trial of NPWT in paediatric burn care.

## Supplementary Information

**Additional file 1.** SPIRIT Checklist: Recommended items to address in a clinical trial protocol and related documents.

**Additional file 2.** Consent form for participants.

## Data Availability

Not applicable for protocol manuscript.

## Abbreviations

NPWT: Negative pressure wound therapy
TBSA: Total body surface area
QoL: Quality of life
RCT: Randomised control trial
PLCBC: Pegg Leditschke Children’s Burns Centre
ED: Emergency Department
PICU: Paediatric Intensive Care Unit
FPS-R: Faces Pain Scale Revised
P-NRS: Pain Number Ranking Scale
FLACC: Face, legs, activity, cry, consolability
I-NRS: Itch Number Ranking Scale
BBSIP: Brisbane based scar impact profile
LDI: Laser Doppler imaging
