# Supplementary material for: The feasibility of negative pressure wound therapy versus standard dressings in paediatric hand and foot burns protocol: a pilot, single-centre, randomised control trial
Source: Pilot Feasibility Stud. 2023 May 26;9:90. doi: 10.1186/s40814-023-01308-z (PMC10214590; doi:10.1186/s40814-023-01308-z)
Supplement: Supplementary file 2 — Additional file 2. Consent form for participants. [file 40814_2023_1308_MOESM2_ESM.docx]

**CONSENT FORM: ENROLMENT TO STUDY**

| **Title** |
| --- |

|  | \| *“The Use of Negative Pressure Wound Therapy in Acute Paediatric Hand and Feet Burns: A Pilot, Prospective Randomised Control Trial”.* \| \| --- \| |
| --- | --- | --- |
| **Edition** | Version 3.0 29/03/2022 |
| **Principal Investigator** | Dr Emma Lumsden, Dr Bronwyn Griffin, Prof Roy Kimble, Ms Kristen Storey,  Ms Catherine McMillan  Pegg Leditschke Children’s Burns Centre,  Queensland Children’s Hospital, South Brisbane, 4101, T: (07) 3069 7392 |

**Declaration by Parent/Guardian**

**It’s okay to say ‘No’**

- I have read the Participant Information Sheet or someone has read it to me in a language that I understand.
- I understand the purposes, procedures and risks of the research described in the project.
- I have had an opportunity to ask questions and I am satisfied with the answers I have received.
- I freely agree to the child participating in this research project as described and understand that I am free to withdraw them at any time during the project without affecting their future health care.
- I freely agree to the child’s research data being used for future purposes.
- I understand that I will be given a signed copy of this document to keep.

|  |  | |  | | | |  |
| --- | --- | --- | --- | --- | --- | --- | --- |
|  | Name of Child (please print) |  | | | | |  |
|  |  |  | | | | |  |
|  | Name of Parent/Guardian (please print) | | |  | | |  |
|  |  | | |  | | |  |
|  | Signature of Parent/Guardian | |  | | Date |  |  |
|  | | | | | | | |

**Declaration by Investigator**

I have given a verbal explanation of the research project, its procedures and risks and I believe that the parent/guardian of the participant has understood that explanation. I have provided the parent/guardian with a copy of the participant information sheet.

|  | | | | | | |
| --- | --- | --- | --- | --- | --- | --- |
|  | Name of Investigator (please print) | |  | | |  |
|  | | | | | |  |
|  | Signature |  | | Date |  |  |
|  | | | | | | |

**Declaration by Independent Witness**

I have witnessed the receipt of a Patient Information Sheet by the parent/guardian and exchanging of information between the investigator and the parent/guardian about the study.

|  | | | | | | |
| --- | --- | --- | --- | --- | --- | --- |
|  | Name of Witness (please print) | |  | | |  |
|  | | | | | |  |
|  | Signature |  | | Date |  |  |
|  | | | | | | |

Note: All parties signing the consent section must date their own signature.

**CONSENT FORM: WITHDRAWAL OF PARTICIPATION**

**Declaration by Parent/Guardian**

| **Title** | \| *“The Use of Negative Pressure Wound Therapy in Acute Paediatric Hand and Feet Burns: A Pilot, Prospective Randomised Control Trial”.* \| \| --- \| |
| --- | --- | --- |
| **Edition** | Version 2.0 3/08/2021 |
| **Principal Investigator** | Dr Emma Lumsden, Dr Bronwyn Griffin, Prof Roy Kimble, Ms Kristen Storey,  Ms Catherine McMillan  Pegg Leditschke Children’s Burns Centre,  Queensland Children’s Hospital, South Brisbane, 4101 T: (07)3069 7392(07) 3069 7392 |

I wish to withdraw the child from participation in the above research project and understand that such withdrawal will not affect their routine treatment, relationship with those treating them or relationship with The Queensland Children’s Hospital.

|  |  | |  | | | |  |
| --- | --- | --- | --- | --- | --- | --- | --- |
|  | Name of Child (please print) |  | | | | |  |
|  |  | |  | |  |  |  |
|  | Name of Parent/Guardian (please print) | | |  | | |  |
|  |  | | |  | | |  |
|  | Signature of Parent/Guardian | |  | | Date |  |  |
|  | | | | | | | |

**Declaration by Investigator^†^**

I have given a verbal explanation of the implications of withdrawal from the research project and I believe that the parent/guardian of the participant has understood that explanation.

|  | | | | | | |
| --- | --- | --- | --- | --- | --- | --- |
|  | Name of Investigator^†^ (please print) | |  | | |  |
|  | | | | | |  |
|  | Signature |  | | Date |  |  |
|  | | | | | | |
|  | | | | | | |

^†^ A member of the research team must provide the explanation of, and information concerning, withdrawal from the research project.

**Declaration by Independent Witness**

I have witnessed the receipt of a Patient Information Sheet by the parent/guardian and exchanging of information between the investigator and the parent/guardian about the study.

|  | | | | | | |
| --- | --- | --- | --- | --- | --- | --- |
|  | Name of Witness (please print) | |  | | |  |
|  | | | | | |  |
|  | Signature |  | | Date |  |  |
|  | | | | | | |

Note: All parties signing the withdrawal section must date their own signature.

**DEMOGRAPHIC DATA: CONSENT FORM**

**Declaration by Parent/Guardian**

| **Title** | \| *“The Use of Negative Pressure Wound Therapy in Acute Paediatric Hand and Feet Burns: A Pilot, Prospective Randomised Control Trial”.* \| \| --- \| |
| --- | --- | --- |
| **Edition** | Version 2.0 03/08/2021 |
| **Principal Investigator** | Dr Emma Lumsden, Dr Bronwyn Griffin, Prof Roy Kimble, Ms Kristen Storey,  Ms Catherine McMillan  Pegg Leditschke Children’s Burns Centre,  Queensland Children’s Hospital, South Brisbane, 4101 T: (07)3069 7392 |

I do not consent to the child’s participation in this study although I freely agree to the child’s demographic information being used in relation to this study. I understand that this information is used to compare information for children participating and children not participating in the study.

|  |  | |  | | | |  |
| --- | --- | --- | --- | --- | --- | --- | --- |
|  | Name of Child (please print) |  | | | | |  |
|  |  | |  | |  |  |  |
|  | Name of Parent/Guardian (please print) | | |  | | |  |
|  |  | | |  | | |  |
|  | Signature of Parent/Guardian | |  | | Date |  |  |
|  | | | | | | | |

**Declaration by Investigator^†^**

I have given a verbal explanation of the implications of withdrawal from the research project and I believe that the parent/guardian of the participant has understood that explanation.

|  | | | | | | |
| --- | --- | --- | --- | --- | --- | --- |
|  | Name of Investigator^†^ (please print) | |  | | |  |
|  | | | | | |  |
|  | Signature |  | | Date |  |  |
|  | | | | | | |

^†^ A member of the research team must provide the explanation of, and information concerning, use of demographic data within the research project.

**Independent Witness**

I have witnessed the receipt of a Patient Information Sheet by the parent/guardian and exchanging of information between the investigator and the parent/guardian about the study.

|  | | | | | | |
| --- | --- | --- | --- | --- | --- | --- |
|  | Name of Witness (please print) | |  | | |  |
|  | | | | | |  |
|  | Signature |  | | Date |  |  |
|  | | | | | | |

Note: All parties signing the withdrawal section must date their own signature.

**NPWT in Paediatric Hand and Feet Burns: Patient Log**

**Recruitment log**

**Patient Name:** ______________

**Age**: ____

**Gender:** M / F

**Date of Injury**:___/____/____,

**Patient recruited:** Yes/No.

**If no, what was the reason:**

Ineligible

Decline consent

Medical decline
